# Tracking crop varieties using genotyping-by-sequencing markers: a case study using cassava (*Manihot esculenta* Crantz)

**DOI:** 10.1186/s12863-015-0273-1

**Published:** 2015-09-23

**Authors:** Ismail Y. Rabbi, Peter A. Kulakow, Joseph A. Manu-Aduening, Ansong A. Dankyi, James Y. Asibuo, Elizabeth Y. Parkes, Tahirou Abdoulaye, Gezahegn Girma, Melaku A. Gedil, Punna Ramu, Byron Reyes, Mywish K. Maredia

**Affiliations:** International Institute of Tropical Agriculture (IITA), PMB 5320 Ibadan, Nigeria; Council for Scientific and Industrial Research-Crops Research Institute (CSIR-CRI), P.O. Box 3785, Kumasi, Ghana; Agriculture Innovation Consult, Kumasi, Ghana; Cornell University, Institute for Genomic Diversity, 175, Biotechnology Building, Ithaca, NY 14853 USA; International Center for Tropical Agriculture (CIAT), Planes de Altamira, de Pizza Hut Villa Fontana, 1c Oeste, Edificio CAR III, Oficina 4-1, Managua, Nicaragua; Michigan State University, 446 W. Circle Drive, Room 89, East Lansing, MI 48824 USA

**Keywords:** Cassava, Variety identification, Impact assessment, Genotyping-by-sequencing, Ancestry estimations

## Abstract

**Background:**

Accurate identification of crop cultivars is crucial in assessing the impact of crop improvement research outputs. Two commonly used identification approaches, elicitation of variety names from farmer interviews and morphological plant descriptors, have inherent uncertainty levels. Genotyping-by-sequencing (GBS) was used in a case study as an alternative method to track released varieties in farmers’ fields, using cassava, a clonally propagated root crop widely grown in the tropics, and often disseminated through extension services and informal seed systems. A total of 917 accessions collected from 495 farming households across Ghana were genotyped at 56,489 SNP loci along with a “reference library” of 64 accessions of released varieties and popular landraces.

**Results:**

Accurate cultivar identification and ancestry estimation was accomplished through two complementary clustering methods: (i) distance-based hierarchical clustering; and (ii) model-based maximum likelihood admixture analysis. Subsequently, 30 % of the identified accessions from farmers’ fields were matched to specific released varieties represented in the reference library. *ADMIXTURE* analysis revealed that the optimum number of major varieties was 11 and matched the hierarchical clustering results. The majority of the accessions (69 %) belonged purely to one of the 11 groups, while the remaining accessions showed two or more ancestries. Further analysis using subsets of SNP markers reproduced results obtained from the full-set of markers, suggesting that GBS can be done at higher DNA multiplexing, thereby reducing the costs of variety fingerprinting. A large proportion of discrepancy between genetically unique cultivars as identified by markers and variety names as elicited from farmers were observed. Clustering results from *ADMIXTURE* analysis was validated using the assumption-free Discriminant Analysis of Principal Components (DAPC) method.

**Conclusion:**

We show that genome-wide SNP markers from increasingly affordable GBS methods coupled with complementary cluster analysis is a powerful tool for fine-scale population structure analysis and variety identification. Moreover, the ancestry estimation provides a framework for quantifying the contribution of exotic germplasm or older improved varieties to the genetic background of contemporary improved cultivars.

**Electronic supplementary material:**

The online version of this article (doi:10.1186/s12863-015-0273-1) contains supplementary material, which is available to authorized users.

## Background

Agricultural productivity in developing countries is affected by limited access to improved varieties, in addition to biotic, abiotic constraints and sub-optimal agronomic practices [1, 2]. Successful dissemination and adoption of improved varieties from both private and public breeding programs is expected to contribute positively to farm-level productivity and income generation. It is the role of household level impact assessment studies, particularly collection of variety specific adoption data, to determine whether this is happening [3, 4].

Traditionally, estimation of improved variety adoption in socio-economic impact studies relies mostly on: expert opinion of breeders, extension services and other experts; elicited responses from farmers in farmer-level surveys; and morphological descriptors. However, such methods have several inherent uncertainty levels. For example, variety naming systems in the absence of formal seed systems can be quite temporally and spatially variable leading to inconsistencies in the names of a particular variety. Also, environmental conditions and different stages of plant development influence morphological descriptors [5, 6]. Finally, the number of descriptors can be quite limited as varieties are developed to conform to desired ideotypes, thus greatly reducing the power to distinguish consanguineous varieties [7].

These challenges can be overcome by using molecular markers which are not only unaffected by the environmental factors and crop developmental stages but are also ubiquitous throughout plant genomes. Genome-wide markers, like single nucleotide polymorphisms (SNP), not only facilitate germplasm classification using genetic distance estimates but can also be used to quantify the relative proportion of ancestries derived from various founder genotypes of currently grown cultivars [8]. Such inferences of ancestries are useful in understanding and/or reconstructing the evolution of successful varieties, either landraces or products of formal breeding programs that lack breeding pedigree records or where the varieties are derived from open-pollinated breeding methods [9]. In the context of impact assessment of a specific breeding program, ancestry inferences can be useful in estimating the benefits resulting from the usage of its improved germplasm by other programs [10]. This is because improved germplasm often moves easily throughout the network of plant breeding systems, resulting in research spill-over benefits.

In the past, simple sequence repeats and anonymous markers such as amplified fragment length polymorphisms and randomly amplified DNA polymorphisms have been used in DNA-based fingerprinting applications [11]. However, due to inadequacies of these markers, including limited multiplexing ability, high genotyping costs and low frequency in the genome, they are increasingly being displaced by SNP markers generated from next-generation sequencing using reduced representation library (RRLs) methods. These recent methods rely on restriction enzymes to target a specific and reproducible subset of the genome for sequencing, thus allowing for simultaneous discovery and scoring of large numbers of markers. Genotyping-by-sequencing [12] is an RRL method that is relatively simple and inexpensive, making it feasible to genotype large populations of individuals. GBS has therefore become very popular, particularly for researchers working on non-model species with limited genomic resources [13].

Here, we report the use of GBS markers for cultivar identification with the objective of tracking released varieties in farmers’ fields, using cassava (*Manihot esculenta* Crantz) as a case study. Cassava is a highly heterozygous, clonally propagated species that originates from Latin America [14]. Its starchy storage roots are the main source of calories for over 500 million people in the tropics [15]. Africa is currently the leading producer of the crop accounting for more than 50 % of global production [16]. Its ability to produce reasonable yields in marginal environments, its tolerance to drought and poor soils, and its ability for in-ground storage to allow piece-meal harvesting makes cassava one of the most important food-security crops in the continent [17]. Despite its importance, planting materials are predominantly sourced from the informal seed system often from the farmer’s own harvest or exchange between farmers [18]. Dissemination of new varieties has often been limited to efforts by the extension services connected to national programs and informal diffusion through farmer-to-farmer exchanges. This situation contributes to the challenge of tracking the spread of such varieties.

## Methods

**Fig. 1:**
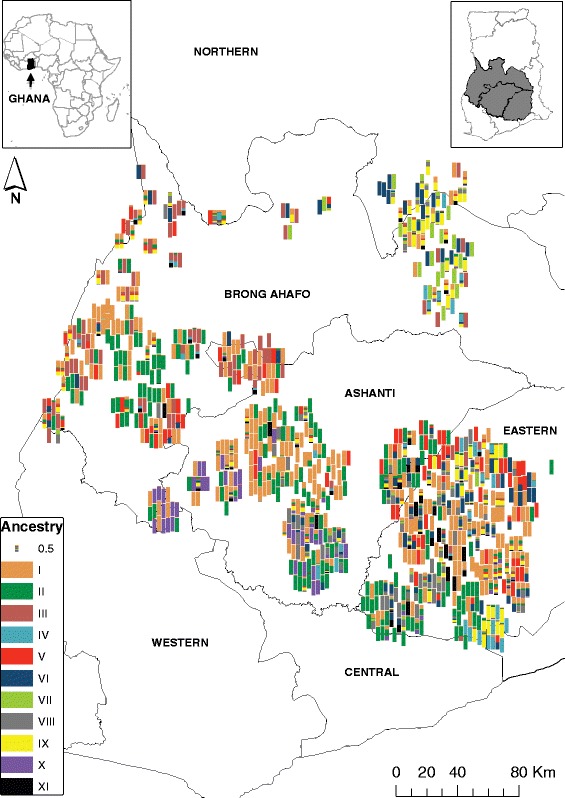
Geographical distribution of the cassava cultivars (landraces and released varieties) analyzed in this study. The color scheme matches that of ancestry assignment in Fig. 3a. Twenty-nine accessions that lacked latitude and longitude information are not shown on the map. Left inset is the overview map of Africa showing the location of Ghana (dark shade) and the right inset shaded grey highlights the three study regions

This study was conducted in three regions of Ghana covering the largest cassava producing area accounting for 61 % of cassava production in the country in 2010 [16, 19]. The three study regions included Brong Ahafo, Ashanti and Eastern (Fig. 1). A total of 495 households were selected using a multi-stage cluster sampling method. These households were distributed across 100 villages from 20 districts in the three study regions. For each of the 495 households surveyed, field sample collection entailed visiting one cassava field for each household with the largest number of varieties. A consent statement was read to the main decision maker of the household to inform him/her about the purpose of the study and to seek his/her permission to visit the cassava field to collect the leaf samples. Data and sample collection proceeded only if the farmer gave the consent. The GPS coordinates of the field were taken and farmers were asked to identify plants representing each of the varieties grown. Apical leaf samples were collected from one plant representing each variety and preserved in silica gel for transportation to a central laboratory at IITA in Ibadan, Nigeria for DNA extraction. Since a major objective of the present study was to identify specific cultivars in farmers’ fields, a “reference library” consisting of 64 clones representing released varieties (*n* = 16) and key landraces (*n* = 48) maintained by the Council for Science and Industrial Research-Crops Research Institute (CSIR-CRI) of Ghana were genotyped alongside the accessions from farmers’ fields. It should be noted that many of the released varieties in Ghana are landraces with superior agronomic traits (resistance to cassava mosaic disease, high yield and dry-matter content) and culinary qualities (root friability after boiling). These landraces were officially released as varieties following multi-year and -locational testing (Prof. S. Kantanka and Prof. J. P. Tetteh, personal communication).

### DNA extraction and GBS

DNA was isolated from 1045 genotypes, representing 917 accessions collected from farmer’s fields and a library of 64 clones genotyped in duplicate (Additional file 1: Table S1). The Dellaporta method [20] with modifications described in [21] was used for high throughput DNA extraction. For genotyping-by-sequencing library preparation, we chose the ApekI restriction enzyme (recognition site: G|CWCG) that produces less variable distributions of read depth and therefore a larger number of scorable SNPs in cassava [22]. Eleven 96-plex GBS libraries were constructed as described in [12] and sequenced at the Institute of Genomic Diversity at Cornell University using the Illumina HiSeq2500. Raw read sequences were processed through cassava GBS production pipelines developed using TASSEL 5.0 initially generated with about 2500 cassava clones under the NextGen Cassava project (www.nextgencassava.org) [13]; http://www.maizegenetics.net/#!tassel/c17q9). Resulting hapmap files (SNPs) were filtered with minor allele frequency (MAF) of 0.001 and coverage of 10x. SNPs were further processed by removing those with MAF of less than 0.01 and loci with more than 40 % missing data. The remaining missing SNP data-points were then imputed using GLMNET [23].

### Cluster analysis

Identification of the cassava varieties was performed using three complementary clustering approaches: (i) pairwise distance-based hierarchical clustering; (ii) model-based maximum likelihood estimation of individual ancestries from multi-locus SNP genotype datasets using *ADMIXTURE* [24]; and (iii) Discriminant Analysis of Principal Components (DAPC) [25].

In the first approach, a pairwise genetic distance (identity-by-state, IBS) matrix was calculated from 56,489 SNP markers in PLINK [26]. A Ward’s minimum variance hierarchical cluster dendrogram was then built from the IBS matrix using the Analyses of Phylogenetics and Evolution (ape) package [27] implemented in *R* [28]. The critical distance threshold to declare whether two genotypes are identical was empirically determined from the distribution of pairwise distances between duplicated DNAs from 64 samples. This “calibration principle” approach [29] was taken because of the possibility of SNP genotype errors resulting from miscalling some heterozygous SNPs with low sequencing read depth as homozygotes [22].

In the second approach, *ADMIXTURE* analysis using the same set of 56,489 SNP markers was used to identify ancestries of the sampled cassava accessions. The number of sub-populations, K, was varied from 2 to 18 (*K*, in this case are considered founders of the currently cultivated varieties in the study regions). The most appropriate K value was selected after considering (i) 10-fold cross-validations whereby the best K exhibits low cross-validation error compared to other K values [30] and (ii) good correspondence with the clustering pattern obtained by the hierarchical tree.

To develop smaller sets of ancestry informative markers (AIMs) for follow-up studies using lower density genotyping, further *ADMIXTURE*–based ancestry estimation was carried out using decreasing subsets of SNP markers. These were selected based on Weir and Cockerham [31] F_ST_, a measure of differences in allele frequencies among the subpopulations detected by *ADMIXTURE*. For comparative purposes, equivalent numbers of markers were randomly selected, each twenty times with replacement. The objective here was to see how much we can reduce the number of markers while still obtaining cluster assignment results that is close to that obtained from the full set of markers. We used the ‘supervised’ *ADMIXTURE* method assuming K = 11 [32]. Accuracies of ancestry estimates was determined through correlations between the subsets and the complete set of 56,489 markers.

The model-based clustering approach implemented in *ADMIXTURE* assumes linkage equilibrium among loci and Hardy-Weinberg equilibrium within ancestral populations [33]. However, such assumptions may be violated in vegetatively propagated species like cassava due to presence of clonal duplicates in germplasm collections. To validate the clustering pattern obtained from *ADMIXTURE* and the hierarchical clustering algorithms, we carried out Discriminant Analysis of Principal Components (DAPC), an assumption-free multivariate clustering method [25] using the R package ‘adegenet’ [34] in a two-step process. Firstly, the optimal number of clusters was inferred using k-means analysis [35] of PCA-transformed genome-wide SNP data. After varying possible number of clusters from 2 to 40, Bayesian Information Criterion (BIC) was used to assess the best supported model i.e. the number and nature of clusters. Secondly, DAPC [25] was carried out on the clusters identified through k-means using the first 70 principal components. Membership probabilities of each individual for the different groups, akin to the sub-population membership coefficients from *ADMIXTURE* was obtained from DAPC. The results of DAPC analysis was then compared with those achieved from *ADMIXTURE*.

## Results

### Field sampling

**Fig. 2:**
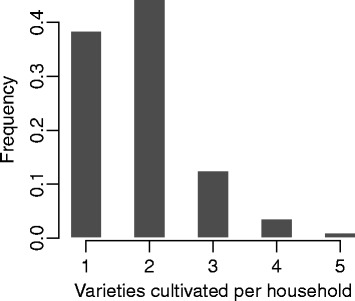
Histogram showing the distribution of the number of varieties cultivated per household

Field surveys found that farmers cultivated between one and five different varieties of cassava in their fields, but majority of them (>80 %) grow only one or two varieties (Fig. 2). A large number of unique farmer-elicited variety names (180) were associated with the 917 accessions collected from the three study regions of Ghana. Most of these names occurred five or less instances in the survey. The two most frequent names were “Debor” and “Ankra”, each recorded 90 and 87 times, respectively.

### Variety identification

An average genetic distance between repeat genotyping of the 64 accessions in the “reference library” was below 0.05 (Ward’s distance, Additional file 2: Figure S1). We therefore chose 0.05 as the distance threshold below which we can declare that two accessions represent the same clone. The residual distance between same DNA is most likely due to miss-calling of heterozygotes as homozygotes from low sequencing read-depth, as is typical in high-multiplexing, sequence-based genotyping methods [22].

**Fig. 3:**
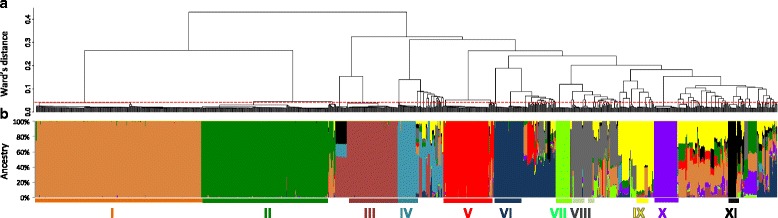
Population structure of cassava accessions from three major cassava producing regions of Ghana. **a** Hierarchical clustering (Ward’s minimum variance method) dendrogram. The red dashed line represents the empirically determined distance threshold developed from comparison of duplicated library samples. A distance of 0.05 below which two individuals can be declared identical. **b** Individual ancestry estimated from *ADMIXTURE* analysis. Individuals are represented as thin vertical lines partitioned into segments corresponding to the inferred membership in K = 11 genetic clusters as indicated by the colors. The roman numerals show groups of clonal individuals with predominant ancestry membership in each of the 11 clusters

Genetic relationships among the 1045 genotyped accessions is described using a hierarchical clustering dendrogram (Fig. 3a) while the estimated ancestries (Q) obtained from *ADMIXTURE* are presented as a barplot (Fig. 3b). Major as well as minor clusters of genetically identical genotypes with genetic distances below the empirically determined distance threshold are clearly discernible. The two most dominant varieties (Cluster I and II) belong to the same branch of the dendrogram, and are therefore likely to share some common farmer-preferred characteristics. According to farmers’ naming system, the first variety which is associated with the most commonly recorded cultivar names (‘Debor’, ‘Ankra’ and ‘Bankye Kokoo’), is quite popular because of its excellent culinary traits, two of which are mealiness after boiling and relatively sweet taste. The remaining clones form a second large cluster that further subdivides into about nine clusters.

**Fig. 4:**
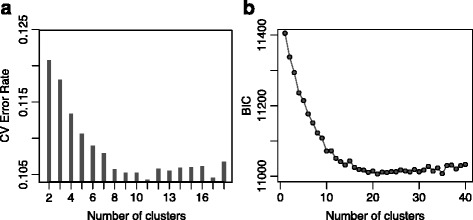
Determination of the optimal number of clusters using *ADMIXTURE* and DAPC. **a** Ten-fold cross-validation error rates for K = 2 to K = 18, showing the least error rate was produced by K = 11. **b** Bayesian Information Criterion (BIC) estimates for k-means clusters (K = 1 to K = 40) in the same dataset

After elucidation of these groupings through hierarchical clustering, we turned to the *STRUCTURE*-like analysis [36] using the *ADMIXTURE* program [24] to assign individuals proportionally to hypothetical founder populations. After varying the number of sub-populations (*K*) from 2 to 18, the most appropriate number was found to be K = 11, which produced the lowest 10-fold cross-validation error compared to other K values (Fig. 4). These groupings corresponded to the hierarchical clustering dendrogram: each of the major branches of the dendrogram formed a distinct ancestry group.

Groups of clones with predominant ancestry membership to one of each of the identified *ADMIXTURE* sub-populations (>90 %) were discernible with the exception of group IX that had small admixture from groups II and III (Fig. 3b). The results of *ADMIXTURE*-based clustering is strongly supported by the topology of the distance-based dendrogram, with most of these genotypes also having very low IBS distance within their respective clusters (Fig. 3a). A large number of individual cassava genotypes (*n* = 277) that share ancestry between two or more of the identified varieties were also detected. Of these, about 157 had at least 50 % of their ancestry coming from one of the eleven sub-populations, while 120 accessions have multiple ancestries (Table 1). Moreover, the proportions of ancestries in these varieties appear to be consistent with simple crossing or backcrossing to produce F1 hybrids or backcross hybrids that may have occurred either in farmer’s fields or in formal plant breeding programs. Other genotypes show more complex multi-parent ancestries.

**Table 1:** Summary of the results of variety identification efforts in the present study

Varieties*	Number of accessions **	Variety Status	Common local names or released variety name (according to CSIR-CRI library)***
Variety I	208	Landrace	Ankra, Bankye kokoo, Debor
Variety II	158	Released variety	IFAD, UCC
Variety III	65	Released variety	Nkabom
Variety IV	17	Released variety	Afisiafi
Variety V	57	Landrace	Akosua tumtum, Bankye tumtum, Tuaka
Variety VI	37	Landrace	Bankye kakaduro, Navrongo
Variety VII	20	Not in library	Ampenkyene
Variety VIII	21	Released variety	Bankye broni
Variety IX	13	Landrace	Gbezeh
Variety X	33	Not in library	Kotee
Variety XI	11	Not in library	Amapomaa
50 % ancestry from Variety I	17	Not in library	Many (12 different names)
50 % ancestry from Variety II	11	Not in library	Many (7 different names)
50 % ancestry from Variety III	19	Released variety	Tek bankye and Dokuduade (12 and 4 accessions, respectively)
50 % ancestry from Variety IV	10	Most not in library	Many (12 different names)
50 % ancestry from Variety V	12	Not in library	Many (10 different names)
50 % ancestry from Variety VI	33	Not in library	Many (25 different names)
50 % ancestry from Variety VIII	21	Not in library	Many (19 different names)
50 % ancestry from Variety IX	29	Not in library	Many (17 different names)
50 % ancestry from Variety XI	5	Not in library	Many (6 different names)
Multi-ancestry clones	120	Most not in library	Sikabankye (Only 2 accessions)

*Admixture analysis-based ancestry estimates show there were 11 major varieties as well as hybrids derived from these varieties. We grouped these as (i) those that have at least 50 % ancestry from each of the major 11 groups and (ii) those that have multiple ancestries with none meeting the 50 % threshold

**For the admixed clones (i.e. hybrids), the numbers designate the totality of the accessions that have at least 50 % of their ancestries coming from a specific genotype

***Because of the multiplicity of names associated with each unique landrace, we only attempt to provide most common ones where applicable

Following clustering of accessions into groups of genetically identical clones, actual variety identities were determined by matching each accession to the samples in the CSIR-CRI library. The library contained a total of 64 accessions but based on genetic similarity, these were collapsed into 34 unique cultivars (Additional file 2: Figure S1) of which 16 are released varieties. Using this library, we successfully classified a total of 282 accessions from the farmers’ fields as released varieties, representing about 30 % of the sampled 917 accessions (Table 1). These accessions matched only 8 of the 16 released varieties in CSIR-CRI reference library. Of the identified varieties, the most common was “IFAD”, also known as “UCC” and found in 158 households. The next most common variety was “Nkabom” (*n* = 65), followed by less common varieties of “Afisiafi”, “Tek Bankye”, “Bankye Broni” and “Doku Duade”, which occurred in 17, 12, 21 and 4 households, respectively. Although “Nkabom” is a released variety in the CSIR-CRI library, it was found to correspond to a superior landrace from Nigeria (TMEB3), one of the first clones discovered to harbor dominant resistance to cassava mosaic disease [37]. It is therefore likely to have been introduced to Ghana through formal germplasm exchange between public breeding programs of the two countries. The least common of the released varieties was “Sika Bankye” found in only two of the surveyed households.

Besides the released varieties, a total of 315 accessions belonging to five different landraces with corresponding clones in the CSIR-CRI library were identified (Table 1). However, we could not match a total of 202 accessions from farmers’ fields to any of the genotypes in the reference library. These belonged to groups VII, X and XI as well as the various hybrid groups (Table 1).

### Geographical distribution of the identified varieties

To further place the results from cluster analyses in a geographical context, we projected individual accessions on the map of Ghana (Fig. 1) using the associated GPS co-ordinates. Each accession is represented by a barplot that is colored according to the inferred membership in the K = 11 genetic clusters. The two most common varieties (I and II) are equally well distributed across the three study regions suggesting they are highly preferred by most farmers and have broad adaptation. On the other extreme, varieties VII and X are geographically restricted and found only in one geographic area.

Variation in the geographic distribution of the released varieties was observed in the three study regions. The most common released variety (Variety II in Fig. 1) is well distributed across the three study regions; Variety III was found mostly in the Brong Ahafo region and a few places in the Eastern Region; Variety IV and VIII occurred mostly in the Eastern Region and in small patches of the other regions (Fig. 1). Potential reasons for the geographic clustering of varieties include region specific uses and adaptation as well as being newly evolved or introduced varieties with limited dissemination opportunities. The location and limited number of industrial processing facilities may also restrict distribution of high yielding varieties suitable for processing.

### Correspondence between local names and each of the identified cultivars

**Fig. 5:**
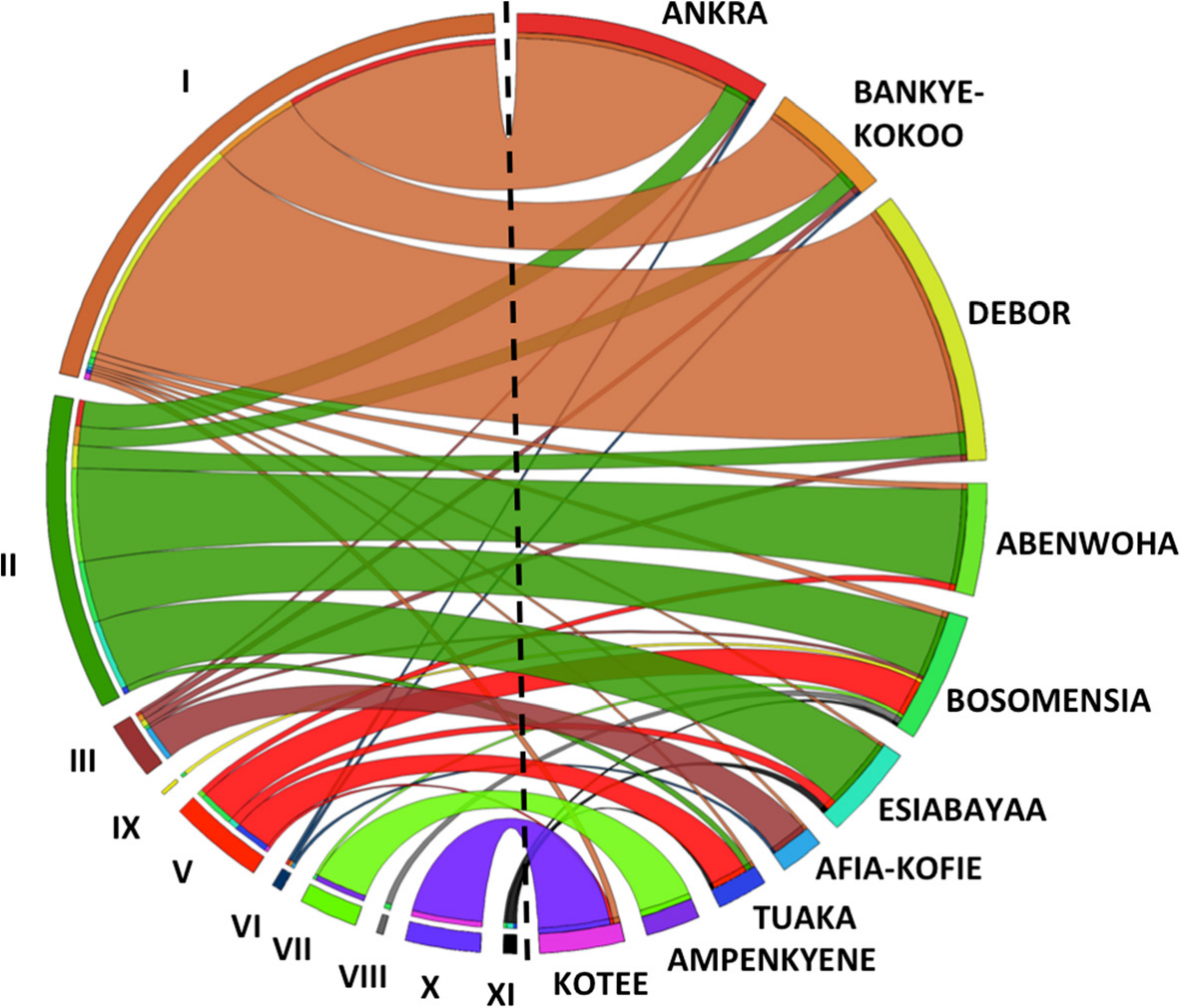
Correspondence between genetically unique varieties as identified by 56,489 SNP (indicated by numbers I to XI on the left semi-circle) and the most common variety names as elicited from farmers (indicated by A to J on the right semi-circle). Note that only variety names occurring at least 10 times or more in the entire sample were used

While many farmer given variety names correspond to specific clones, there are often differences between genetically unique cultivars as identified by 56,489 SNPs and variety names as elicited from farmers (Fig. 5). For example, the most common clone (Variety I) was variously named as “Debor”, “Bankye Kokoo” and “Ankra”, as well as other less common names not shown in Fig. 5. Spatial distribution analysis revealed that these three most commonly used names are geographically structured by regions (Additional file 3: Figure S2) suggesting there are regional differences in the name of the same variety. The naming system was similarly complex for the eight released varieties cultivated by farmers. For instance, the two most common released varieties (IFAD/UCC and Nkabom) were associated with 33 and 25 different names, respectively (Additional file 1: Table S1). Such discrepencies resulting from synonymy and homonymy in clones names is expected to confound tracking of released varieties when relying on use of names alone.

### Performance of reduced numbers of SNP markers in cultivar identification

**Fig. 6:**
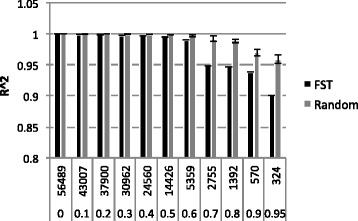
Average accuracy (R^2^) and standard deviation (error bars) of *ADMIXTURE*-based estimation of individual ancestries using: i) SNP panels selected according to increasing F_ST_ thresholds compared with; ii) same number of randomly selected markers. The accuracy was estimated by correlating the ancestry estimates from the various subsets with that obtained using the entire marker data

Accuracy of ancestry estimates (mean R^2^ and SD) obtained with subsets of SNP markers, selected according to increasing F_ST_ (0.1, 0.2,…, 0.9, and 0.95), the corresponding equivalent numbers of randomly sampled SNPs, and the full set of 56,489 SNPs are presented in Fig. 6. Markers passing the predetermined F_ST_ thresholds were the complete set of SNPs, 43007, 37900, 30962, 24560, 14426, 5359, 2755, 1392, 570 and 324, respectively. We found that the randomly drawn SNP panels slightly but consistently outperformed the SNP panels selected according to F_ST_ (average R^2^ = 0.97 for F_ST_ and R^2^ = 0.99 for the random SNPs). In addition, each of the 20 independently drawn samples for each subset produced very similar results, as indicated by the small standard deviation. A very high correlation with the full SNP data was obtained using SNPs with F_ST_ below 0.6 (5359 SNP) and both random and F_ST_-based subsets performed similarly. Using 2755 SNPs (F_ST_ > 0.70) resulted in (0.05 units) lower correlations. The accuracy of 324 SNPs with F_ST_ > 0.95 was substantially lower (R^2^ = 0.90), even much less than that obtained from a similar number but randomly drawn SNPs (R^2^ = 0.96). Our results suggest there is loss of information in predicting admixture when going for markers with larger F_ST_ while random samples of SNPs give higher accuracies, though the actual differences are small. New SNPs-based variety identification studies for cassava would therefore require at least 300 informative SNP markers in order to have sufficient power to not only identify varieties but also estimate ancestries of these accessions.

### Cluster analysis using DAPC and validation of ADMIXTURE results

We validated maximum likelihood-based clustering results from *ADMIXTURE* analysis using DAPC method that is considered free of Hardy-Weinberg and linkage disequilibrium assumptions. Model selection using BIC revealed the presence of hierarchical structure in the population, with steep decline from K = 2 up to around K = 10 followed by a more gentle decrease. The lowest BIC value which corresponded to optimal cluster number was obtained at K = 21 (Fig. 4b). Although this number was larger than that found by *ADMIXTURE* (K = 11), DAPC clustering recapitulated the groupings uncovered by both the distance-based hierarchical clustering topology as well as ancestry estimates achieved by *ADMIXTURE*. Comparison of the cluster membership results from the DAPC and *ADMIXTURE* analyses are summarized in Additional file 4: Table S2. A major difference between the results of the two clustering methods was the propensity of the DAPC analysis to assigned entire individuals to a single cluster compared to *ADMIXTURE*, which was able to assign admixed individuals to multiple clusters. Indeed, whereas a total of 277 genotypes (or 339 when including the CSIR-CRI library accessions) did not meet the 90 % threshold for belonging to single cluster in the *ADMIXTURE*, only 15 genotypes did not to not exceed the same threshold in the DAPC analysis. Of the accessions belonging purely to clusters I to XI (i.e. ancestry > 90 %) from *ADMIXTURE* analysis, we found 100 % agreement with their corresponding DAPC clusters, except for cluster VI whose members were assigned to two DAPC groups (9 and 14) in roughly equal proportions (Additional file 4: Table S2). Large number clusters from DAPC mostly corresponded to sets of genetically similar groups of admixed individuals that shared same ancestries (Additional file 4: Table S2).

## Discussion

As a clonally propagated crop, cassava has several special characteristics for consideration, which also makes it an interesting crop for this case study. First, due to its broad tropical distribution and its predominantly outbreeding system, cassava carries considerable heterozygosity [38]. As expected in typical subsistence farming systems, a substantial number of cassava farmers cultivate more than one variety in their fields to take care of diverse needs. This multiplicity of varieties in farmers’ fields, enables cross-breeding, and eventually some of the volunteer seedlings end-up being selected either consciously or unconsciously as new varieties that are subsequently exchanged [39]. This is the most likely explanation for the occurrence of numerous admixtures in the study region.

Second, as a so-called “orphan crop” [40], cassava improvement has been mainly implemented by public breeding programs and lacks a formal seed system, thereby making varietal dissemination a challenge [41]. Most farmers use their own planting materials (usually stem cuttings from the preceding crop) or they source stem cuttings from neighboring farmers [18]. Even released varieties may be relatively old due to the low rate of variety turn-over [42]. This allows for spontaneous emergence of clonal variants with different phenotypes that may be undistinguishable by molecular markers.

Genetic distance based on use of molecular markers has been proposed as an appropriate tool to identify putative ‘essentially derived varieties’ [11, 43–45]. The concept of essential derivation is often used in relation to protection of breeder’s rights and refers to a variety with slight modifications from an original variety (such as a single gene insertion through transgenic approaches, back-crossing, or induced mutagenesis) [29]. Our study has followed similar principles, but the objective is different: to assist in collecting accurate variety-specific identification data that can be used to study rates of adoption. However, the success of DNA-based sample identification procedure ultimately depends on the availability of a library panel containing representatives of known varieties. Ideally, the library should be as comprehensive as possible and well curated. In our study, we found instances of accessions from farmers that did not have corresponding genotypes in the library (i.e. Cluster groups VII, X and XI). Moreover, several sets of differently named duplicates were found in the library. Most of these duplicates are classified as landraces and were perhaps independently collected from different regions of Ghana and therefore came with different names.

Next generation sequencing-based genotyping methods such as GBS yield thousands to hundreds of thousands of SNP markers, depending on the genome size, choice of restriction enzymes and the level of sample multiplexing. In the present study, we obtained more than half a million SNPs, which were reduced to about 56,000 markers after curation. Cluster analysis using subsets of either randomly selected or F_ST_-selected SNP markers showed that smaller number of markers could produce similar results to those obtained from complete marker data. An ideal set of ancestry informative SNP markers should have one allele that is fixed in one ancestral lineage and not present in the other [46]. Such sets of markers are designed to provide most of the ancestry information using low density cost-effective SNP genotyping arrays and will be valuable for follow-up studies. Numerous ancestry-specific SNP markers have been developed and used in human population studies [47–49]. However, the number of markers required for population assignment will depend on the populations under consideration, their respective level of genetic differentiation and the desired stringency of assignment. Use of array-based genotyping with a fixed set of pre-selected SNPs thus requires an upfront investment and research to determine the genetic structure of the target study population. A more plausible alternative is to use GBS, which at a higher multiplexing level (for instance 384 DNA samples instead of 96) will be cost-effective enough for direct genotype identification. Although higher multiplexing of samples will proportionately reduce the number of scorable SNPs, it is expected that the final number will still be more than sufficient for cultivar identification. In other words, the increasingly affordable sequencing-based DNA fingerprinting methods should be employed as the primary variety identification tool in collection of variety specific adoption data during household level impact assessment studies.

In the present study, the distance-based approach was successfully used to match accessions from farmers’ fields to corresponding varieties in the ‘library’ of released varieties maintained by CSIR-CRI, based on pairwise distance threshold determined empirically from redundant genotyping of a subset of the collection. We then went further by unraveling the underlying population structure of the studied germplasm with the aim of determining the ancestry of individual accessions. In impact assessment studies, the ancestry information is important since it provides a framework for determining the contribution of specific germplasm in development of new varieties and therefore show indirect impact of germplasm originating from a specific breeding program [50]. This was achieved through the analysis of the populations structure from the high-density SNP data using the complementary model-based methods of *ADMIXTURE* and discriminant analysis of principal components. In the absence of reliable pedigree records or where varieties are selected from open-pollinated seeds, ancestry analysis from DNA markers is the only way uncovering the genetic source of varieties. The DAPC method uncovered more clusters than *ADMIXTURE* (Additional file 4: Table S2) but whereas the latter method revealed large number of individuals with two or more ancestries, DAPC mostly assigned individuals to single clusters. This is because the DAPC approach relies on discriminant functions that seeks to maximize the diversity between clusters by while minimizes within-cluster diversity [25]. Such method works best with discontinuous population structure such as in island-model but was found to be less efficient in cassava germplasm due to their continuous and complex population structure [51, 52]. In clonal crop species like cassava, varieties are often derived from complex inter-generational crosses, resulting in clusters that tend to dissolve into clinal patterns of genetic differentiation [25]. Still, DAPC cluster assignment generally agreed with the main *ADMIXTURE* clusters where >90 % ancestries were assigned to specific clusters. In conclusion, this study confirms the reliability and accuracy of high-density SNP markers from sequencing-based genotyping methods for variety identification and tracking adoption of crop varieties.

## Availability of supporting data

The SNP data sets supporting the result of this article are available at www.cassavabase.org (ftp://ftp.cassavabase.org/manuscripts/Rabbi_et_al_2015.zip).

## Additional files

Additional file 1: Table S1. Sample information associated with the 917 accessions from three regions of Ghana and the library of known varieties from CSIR-CRI. *ADMIXTURE*-based ancestry estimates according to predefined eleven clusters are also provided. We have also attempted to, as far as the library is concerned, classify each of the farmers’ accessions to their matching released varieties in the CSIR-CRI library. (XLSX 151 kb) Additional file 2: Figure S1. Hierarchical clustering dendrogram of the duplicated DNA samples. The dashed red line indicates the threshold for declaring genetic identity (i.e. distance between this threshold is spurious and results from GBS SNP calling error). (PDF 17 kb) Additional file 3: Figure S2. Strong geographical structure associated with the most common names attributed to Variety I. (PDF 114 kb) Additional file 4: Table S2. An overview of the distribution of accessions present in the *ADMIXTURE* clusters and the groups identified by the DAPC. (DOCX 83 kb)

## Abbreviations

AIMs: Ancestry Informative Markers
CSIR-CRI: Council for Scientific and Industrial Research - Crops Research Institute
GBS: Genotyping-By-Sequencing
GPS: Global Positioning System
IITA: International Institute of Tropical Agriculture
MAF: Minor allele frequency
RRL: Reduced Representation Library
SNP: Single Nucleotide Polymorphism
IBS: Identity-By-State
PCA: Principal Component Analysis

## References

[1] Fermont AM, van Asten PJA, Tittonell P, van Wijk MT, Giller KE (2009). Closing the cassava yield gap: an analysis from smallholder farms in east africa. Field Crop Res.

[2] Asfaw S, Kassie M, Simtowe F, Lipper L. Poverty reduction effects of agricultural technology adoption: a micro-evidende from rural Tanzania. J Develop Studies. 2012;48(9):1288––1305.

[3] Morris ML, Tripp R, Dankyi AA (1999). Adoption and impacts of improved maize production technology: a case study of the Ghana grains development project.

[4] Shiferaw B, Kassie M, Jaleta M, Yirga C (2014). Adoption of improved wheat varieties and impacts on household food security in Ethiopia. Food Policy.

[5] Elhoumaizi M, Saaidi M, Oihabi A, Cilas C. Phenotypic diversity of date-palm cultivars(*Phoenix dactylifera* L.) from Morocco. Genet Resour Crop Ev. 2002;49(5):483–90.

[6] Racchi ML, Bove A, Turchi A, Bashir G, Battaglia M, Camussi A (2014). Genetic characterization of Libyan date palm resources by microsatellite markers. *3*. Biotech.

[7] Duminil J, Di Michele M (2009). Plant species delimitation: a comparison of morphological and molecular markers. Plant Biosystems.

[8] Yoon MS, Song QJ, Choi IY, Specht JE, Hyten DL, Cregan PB (2007). BARCSoySNP23: a panel of 23 selected SNPs for soybean cultivar identification. Theor Appl Genet.

[9] Jones AG, Ardren WR (2003). Methods of parentage analysis in natural populations. Mol Ecol.

[10] Morris ML, Heisey PW (2003). Estimating the benefits of plant breeding research: methodological issues and practical challenges. Agr Econ.

[11] Ercisli S, Ipek A, Barut E. SSR marker-based DNA fingerprinting and cultivar identification of olives (*Olea europaea*). Biochem Genet. 2011;49(9–10):555–61. 10.1007/s10528-011-9430-z 21476017

[12] Elshire RJ, Glaubitz JC, Sun Q, Poland JA, Kawamoto K, Buckler ES (2011). A robust, simple Genotyping-by-Sequencing (GBS) approach for high diversity species. PLoS One.

[13] Glaubitz JC, Casstevens TM, Lu F, Harriman J, Elshire RJ, Sun Q, et al. TASSEL-GBS: a high capacity genotyping - by - sequencing analysis pipeline. PLoS One. 2014;9(2), e90346. 10.1371/journal.pone.0090346 PMC3938676 24587335

[14] Olsen KM, Schaal BA. Evidence on the origin of cassava: phylogeography of *Manihot esculenta*. Proc Natl Acad Sci. 1999;96(10):5586–91. 10.1073/pnas.96.10.5586 PMC21904 10318928

[15] FAO (2008). Why cassava?.

[16] FAOSTAT (2014). Statistical database.

[17] Okogbenin E, Setter TL, Ferguson M, Mutegi R, Ceballos H, Olasanmi B (2013). Phenotypic approaches to drought in cassava: review. Front Physiol.

[18] Mtunguja MK, Laswai HS, Muzanila YC, Ndunguru J. Farmer’s Knowledge on Selection and Conservation of Cassava (*Manihot esculanta*) Genetic Resources in Tanzania. J Biol, Agriculture HealthCare 2014;4(10):74–78

[19] Angelucci F. Analysis of incentives and disincentives for cassava in Ghana. Technical notes series. MAFAP, FAO, Rome; 2013

[20] Dellaporta SL, Wood J, Hicks JB (1983). A plant DNA minipreparation Version II. Plant Mol Biol Rep.

[21] Rabbi I, Hamblin M, Gedil M, Kulakow P, Ferguson M, Ikpan AS (2014). Genetic mapping using genotyping-by-sequencing in the clonally propagated cassava. Crop Sci.

[22] Hamblin MT, Rabbi IY. The effects of restriction-enzyme choice on properties of genotyping-by-sequencing libraries: a study in cassava (*Manihot esculenta*). Crop Sci. 2014;54(6):2603–8.

[23] Friedman J, Hastie T, Tibshirani R (2010). Regularization paths for generalized linear models via coordinate descent. J Stat Software.

[24] Alexander DH, Novembre J, Lange K (2009). Fast model-based estimation of ancestry in unrelated individuals. Genome Res.

[25] Jombart T, Devillard S, Balloux F (2010). Discriminant analysis of principal components: a new method for the analysis of genetically structured populations. BMC Genet.

[26] Purcell S, Neale B, Todd-Brown K, Thomas L, Ferreira MA, Bender D (2007). PLINK: a tool set for whole-genome association and population-based linkage analyses. Am J Hum Genet.

[27] Paradis E, Claude J, Strimmer K (2004). APE: analyses of phylogenetics and evolution in R language. Bioinformatics.

[28] R Core Team. R: A language and environment for statistical computing. In*.* Vienna, Austria.URL http://www.R-project.org/. R Foundation for Statistical Computing 2013.

[29] Noli E, Teriaca MS, Conti S (2013). Criteria for the definition of similarity thresholds for identifying essentially derived varieties. Plant Breed.

[30] Alexander DH, Lange K (2011). Enhancements to the ADMIXTURE algorithm for individual ancestry estimation. BMC Bioinformatics.

[31] Weir BS, Cockerham CC (1984). Estimating F-statistics for the analysis of population structure. Evolution.

[32] Liu Y, Nyunoya T, Leng S, Belinsky SA, Tesfaigzi Y, Bruse S. Softwares and methods for estimating genetic ancestry in human populations. Hum Genomics 2013, 7:1. doi:10.1186/1479-7364-7-1 10.1186/1479-7364-7-1 PMC3542037 23289408

[33] Frichot E, Mathieu F, Trouillon T, Bouchard G, Francois O (2014). Fast and efficient estimation of individual ancestry coefficients. Genetics.

[34] Jombart T (2008). adegenet: a R package for the multivariate analysis of genetic markers. Bioinformatics.

[35] Legendre P, Legendre L (1998). Numerical ecology.

[36] Weiss KM, Long JC (2009). Non-Darwinian estimation: my ancestors, my genes’ ancestors. Genome Res.

[37] Akano O, Dixon O, Mba C, Barrera E, Fregene M (2002). Genetic mapping of a dominant gene conferring resistance to cassava mosaic disease. Theor Appl Genet.

[38] Wang W, Feng B, Xiao J, Xia Z, Zhou X, Li P, et al. Cassava genome from a wild ancestor to cultivated varieties. Nat Commun. 2014;5, 10.1038/ncomms6110 10.1038/ncomms6110 PMC4214410 25300236

[39] Duputié A, DeléTre M, De Granville J-J, McKey D. Population genetics of *Manihot esculenta* ssp. *flabellifolia* gives insight into past distribution of xeric vegetation in a postulated forest refugium area in northern Amazonia. Mol Ecol. 2009;18(13):2897–907. 10.1111/j.1365-294X.2009.04231.x 19500251

[40] Fauquet CM, Taylor NJ, Tohme J (2012). The global cassava partnership for the 21^st^ century (GCP21). Trop Plant Biol.

[41] Kyamanywa S, Kashaija I, Getu E, Amata R, Senkesha N, Kullaya A. Enhancing food security through improved seed systems of appropriate varieties of cassava, potato and sweetpotato resilient to climate change in Eastern Africa. Nairobi, Kenya: ILRI; 2011. p. 1–28.

[42] Krishna VV, Spielman DJ, Veettil PC, Ghimire S (2014). An empirical examination of the dynamics of varietal turnover in Indian wheat.

[43] Busti A, Caceres M, Calderini O, Arcioni S, Pupilli F. RFLP markers for cultivar identification in tall fescue (*Festuca arundinacea* Schreb.). Genet Resour Crop Evol. 2004;51(4):443–8.

[44] Wu B, Zhong G-Y, Yue J-Q, Yang R-T, Li C, Li Y-J (2014). Identification of pummelo cultivars by using a panel of 25 selected snps and 12 dna segments. PLoS One.

[45] Rodrigues DH, Neto FdA, Schuster I. Identification of essentially derived soybean cultivars using microsatellite markers. Crop Breed Appl Biotechnol. 2008;8(1):74—78.

[46] Rosenberg NA, Li LM, Ward R, Pritchard JK (2003). Informativeness of genetic markers for inference of ancestry. Am J Hum Genet.

[47] Huckins LM, Boraska V, Franklin CS, Floyd JAB, Southam L, GCAN (2014). Using ancestry-informative markers to identify fine structure across 15 populations of European origin. Eur J Hum Genet.

[48] Kosoy R, Nassir R, Tian C, White PA, Butler LM, Silva G (2009). Ancestry informative marker sets for determining continental origin and admixture proportions in common populations in America. Hum Mutat.

[49] Qin P, Li Z, Jin W, Lu D, Lou H, Shen J (2014). A panel of ancestry informative markers to estimate and correct potential effects of population stratification in Han Chinese. Eur J Hum Genet.

[50] Sawler J, Reisch B, Aradhya MK, Prins B, Zhong G-Y, Schwaninger H (2013). Genomics assisted ancestry deconvolution in grape. PLoS One.

[51] de Oliveira EJ, Ferreira CF, da Silva SV, de Jesus ON, Oliveira GA, da Silva MS (2014). Potential of SNP markers for the characterization of Brazilian cassava germplasm. Theor Appl Genet.

[52] Kawuki RS, Herselman L, Labuschagne MT, Nzuki I, Ralimanana I, Bidiaka M, et al. Genetic diversity of cassava (*Manihot esculenta* Crantz) landraces and cultivars from southern, eastern and central Africa. Plant Genet Resour. 2013;11(02):170–81.

