# Supplementary figures and images for: Tracking crop varieties using genotyping-by-sequencing markers: a case study using cassava (Manihot esculenta Crantz)
Source: BMC Genet. 2015 Sep 23;16:115. doi: 10.1186/s12863-015-0273-1 (PMC4580218; doi:10.1186/s12863-015-0273-1)

# Variety I (TME117)

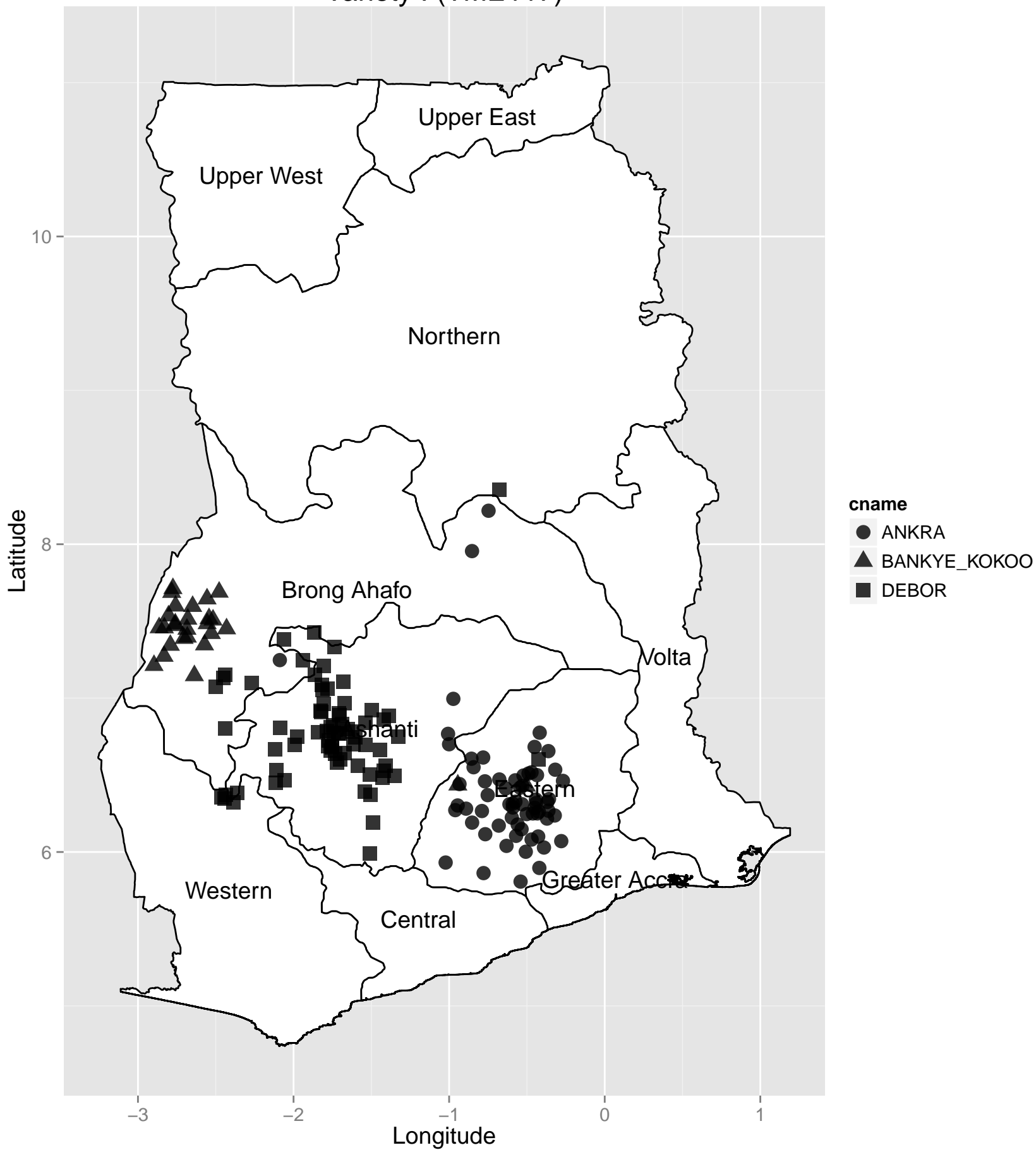

Supplement: Additional file 2: Figure S1. — Hierarchical clustering dendrogram of the duplicated DNA samples. The dashed red line indicates the threshold for declaring genetic identity (i.e. distance between this threshold is spurious and results from GBS SNP calling error). (PDF 17 kb) [file 12863_2015_273_MOESM2_ESM.pdf]

Distance

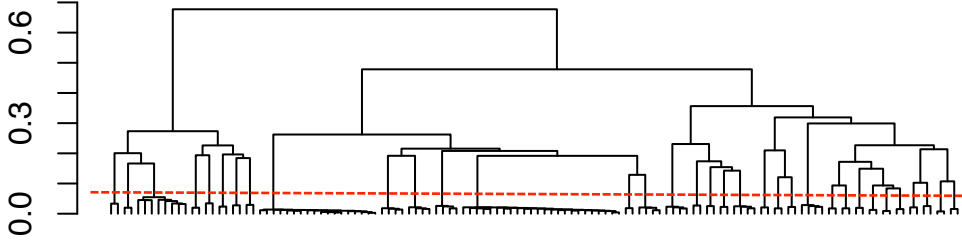

Supplement: Additional file 3: Figure S2. — Strong geographical structure associated with the most common names attributed to Variety I. (PDF 114 kb) [file 12863_2015_273_MOESM3_ESM.pdf]
