# Supplementary material for: Tracking crop varieties using genotyping-by-sequencing markers: a case study using cassava (Manihot esculenta Crantz)
Source: BMC Genet. 2015 Sep 23;16:115. doi: 10.1186/s12863-015-0273-1 (PMC4580218; doi:10.1186/s12863-015-0273-1)
Supplement: Additional file 4: Table S2. — An overview of the distribution of accessions present in the ADMIXTURE clusters and the groups identified by the DAPC. (DOCX 83 kb) [file 12863_2015_273_MOESM4_ESM.docx]

**Supplementary Table 2.** An overview of the distribution of accessions present in the *ADMIXTURE* clusters and the groups identified by the DAPC.

| ADMIXTURE clusters | DAPC clusters | | |  |  |  |  |  |  |  |  |  |  |  |  |  |  |  |  |  |  |
| --- | --- | --- | --- | --- | --- | --- | --- | --- | --- | --- | --- | --- | --- | --- | --- | --- | --- | --- | --- | --- | --- |
|  | 1 | 2 | 3 | 4 | 5 | 6 | 7 | 8 | 9 | 10 | 11 | 12 | 13 | 14 | 15 | 16 | 17 | 18 | 19 | 20 | 21 |
| **I** | - | - | - | - | - | - | - | - | 100 | - | - | - | - | - | - | - | - | - | - | - | - |
| **II** | - | - | - | 100 | - | - | - | - | - | - | - | - | - | - | - | - | - | - | - | - | - |
| **III** | - | - | - | - | - | - | - | - | - | - | - | - | - | - | - | - | - | - | - | - | 100 |
| **IV** | - | - | - | - | - | - | - | - | - | - | - | 100 | - | - | - | - | - | - | - | - | - |
| **V** | - | - | - | - | - | - | - | - | - | - | - | - | - | - | 100 | - | - | - | - | - | - |
| **VI** | - | - | - | - | - | - | - | - | - | 48.8 | - | - | - | 51.2 | - | - | - | - | - | - | - |
| **VII** | - | - | 100 | - | - | - | - | - | - | - | - | - | - | - | - | - | - | - | - | - | - |
| **VIII** | - | - | - | - | - | - | - | 100 | - | - | - | - | - | - | - | - | - | - | - | - | - |
| **IX** | - | - | - | - | - | - | - | - | - | - | - | - | 100 | - | - | - | - | - | - | - | - |
| **X** | - | - | - | - | - | - | 100 | - | - | - | - | - | - | - | - | - | - | - | - | - | - |
| **XI** | - | - | - | - | - | - | - | - | - | - | - | - | - | - | - | - | - | - | 100 | - | - |
| Admix.I | - | - | - | - | - | 11.8 | - | 11.8 | 41.2 | - | - | - | - | - | - | 17.7 | - | 5.9 | 5.9 | 5.9 | - |
| Admix.II | - | - | - | 81.8 | - | - | - | - | - | - | - | - | - | - | - | 18.2 | - | - | - | - | - |
| Admix.III | - | 18.5 | - | - | - | - | - | - | - | - | 59.3 | - | - | - | - | - | - | 11.1 | - | - | 11.1 |
| Admix.IV | - | 60.0 | - | - | - | - | - | - | - | - | - | 10.0 | - | - | - | - | 20.0 | 10.0 | - | - | - |
| Admix.V | - | - | - | - | - | - | - | 8.3 | - | - | - | - | - | - | 50.0 | 8.3 | - | - | - | 33.3 | - |
| Admix.VI | - | - | - | - | - | 12.1 | - | - | - | - | - | - | - | 3.0 | - | - | - | - | - | 84.9 | - |
| Admix.VIII | 3.6 | - | - | - | - | - | - | 92.9 | - | - | - | - | - | - | - | - | - | - | - | 3.6 | - |
| Admix.IX | - | - | - | - | 34.5 | - | - | - | - | - | - | - | 62.1 | - | - | 3.5 | - | - | - | - | - |
| Admix.XI | - | - | - | - | - | - | - | - | - | - | - | - | - | - | - | - | - | - | 66.7 | 33.3 | - |
| Admix.multiple | 13.1 | 7.2 | - | - | 4.6 | 4.6 | - | 9.8 | - | - | - | - | 1.3 | - | - | 37.9 | - | 2.0 | 12.4 | 7.2 | - |
